# Added Sugars and Low- and No-Calorie Sweeteners in a Representative Sample of Food Products Consumed by the Spanish ANIBES Study Population

**DOI:** 10.3390/nu10091265

**Published:** 2018-09-07

**Authors:** María de Lourdes Samaniego-Vaesken, Emma Ruiz, Teresa Partearroyo, Javier Aranceta-Bartrina, Ángel Gil, Marcela González-Gross, Rosa M. Ortega, Lluis Serra-Majem, Gregorio Varela-Moreiras

**Affiliations:** 1 Department of Pharmaceutical and Health Sciences, Faculty of Pharmacy, CEU San Pablo University, 28668 Madrid, Spain; l.samaniego@ceu.es (M.d.L.S.-V.); t.partearroyo@ceu.es (T.P.); 2 Spanish Nutrition Foundation (FEN), 28010 Madrid, Spain; eruiz@fen.org.es; 3 Department of Food Sciences and Physiology, University of Navarra, Pamplona, 31009 Navarra, Spain; javieraranceta@gmail.com; 4 Research Institute of Biomedical and Health Sciences (IUIBS), University of Las Palmas de Gran Canaria, 35016 Las Palmas, Spain; lluis.serra@ulpgc.es; 5 Department of Physiology, Faculty of Medicine, University of the Basque Country (UPV/EHU), 48940 Leioa, Vizcaya, Spain; 6 CIBEROBN, Biomedical Research Networking Center for Physiopathology of Obesity and Nutrition, Carlos III Health Institute, 28029 Madrid, Spain; agil@ugr.es (Á.G.); marcela.gonzalez.gross@upm.es (M.G.-G.); 7 Department of Biochemistry and Molecular Biology II, Institute of Nutrition and Food Sciences, University of Granada, 18010 Granada, Spain; 8 ImFINE Research Group, Department of Health and Human Performance, Universidad Politécnica de Madrid, 28040 Madrid, Spain; 9 Department of Nutrition and Food Science, Faculty of Pharmacy, Madrid Complutense University, 28040 Madrid, Spain; rortega@ucm.es; 10 Service of Preventive Medicine, Complejo Hospitalario Universitario Insular Materno Infantil (CHUIMI), Canary Health Service, Las Palmas de Gran Canaria, 35016 Las Palmas, Spain

**Keywords:** added sugars, low- and no-calorie sweeteners, additives, food groups, processed foods, Spanish population

## Abstract

Low- and no-calorie sweeteners (LNCS), intensely sweet compounds that virtually contain no calories, are used to replace added sugars in food and drinks. Knowledge about different LNCS data in Spanish foods and added sugar sources in Spain is limited, therefore our aim was to identify and compare their presence across main food groups consumed. Food and beverage products (*n* = 434) were obtained from the ANIBES Study (anthropometric data, macronutrients and micronutrients intake, practice of physical activity, socioeconomic data and lifestyles), a cross-sectional study of a representative sample of the Spanish population (9–75 years old; *n* = 2009) carried out in 2013. Food records were obtained from a three-day dietary record using a tablet device. Label data from 1,164 products of different brands were collected and reviewed for content of added sugars and LNCS. LNCS were present in diet soft drinks (100%), “other sweets” (89%), soya drinks (45%), and yogurt and fermented milks (18%). Added sugars were present mainly in sugar soft drinks (100%), energy drinks (96%), sports drinks (96%), bakery and pastry (100%), chocolates (100%), ice cream (100%), breakfast cereals/bars (96%) and jams (89%). Main LNCS were acesulfame K, aspartame, cyclamate and sucralose. Sucrose, dextrose, glucose-fructose syrup, caramel and honey were the main added sugars. Our results show the diversity of foods groups including these ingredients. These data are not compiled in food composition databases, which should be periodically updated to include LNCS and added sugars to facilitate their assessment and monitoring in nutritional surveys.

## 1. Introduction

Over the past decades, it has become evident that an excessive intake of added sugars has many detrimental effects on health, being a contributing factor for increased overweight and obesity rates, higher risk of diabetes and cardiometabolic effects, among others [1]. The World Health Organization (WHO) defines “free sugars” as monosaccharides and disaccharides added to foods and drinks by the manufacturer, cook, or consumer, and sugars naturally present in honey, syrups, fruit juices, and nectar juices [2]. According to the first definition, “free sugars” are similar to added sugars, as the term includes all sugars and syrups added to foods; however, “free sugars” also refers sugars naturally present in fruits. For the purpose of the present work, the term “added sugar” will only apply to those added extrinsically during processing [3]. In 2003, the WHO issued the population nutrient intake goals, which comprised the limitation of free sugar intakes to less than 10% of total energy (TE) [4]. In 2015, WHO published the updated guidelines on free sugar intakes for adults and children in relation to body weight and oral health recommending further reductions [2].

At present, processed food products are one of the main sources of added sugar in our diets and there has been a significant increase in the availability and purchase of these foods in most developed countries including Spain, in which it has almost tripled between 1990 and 2010 (from 11.0% to 31.7%) [5]. The ANIBES Study (anthropometric data, macronutrients and micronutrients intake, practice of physical activity, socioeconomic data and lifestyles), a cross-sectional study of a representative sample of the Spanish population (9–75 years old; *n* = 2009) carried out in 2013, had the objective of updating the main food and beverage group intake and their contribution to the total energy intakes of the population [6]. In this study, results showed that median total sugar intake of participants contributed to 17% of TE intake: 7.3% for added and 9.6% for the intrinsic sugar intake [3]. Furthermore, differences were observed by age groups for added sugars, being highest in children and adolescents. Regarding intrinsic sugars, however, a higher contribution to TE was observed in the elderly. Moreover, 58.2% of children fulfilled WHO recommendations (<10% TE), lower for adolescents (52.6%) and markedly higher in adults (76.7%) and elderly (89.8%) [3].

Low- and no-calorie sweeteners (LNCS) are used as sugar substitutes and their presence in foods and beverages has increased rapidly over the past 30 years [7]. This trend is expected to continue to rise after the publication of a Collaboration Plan for the improvement of the composition of food and beverages, by the Spanish Ministry of Health, Social Services and Equality, through the Spanish Agency for Consumer Affairs, Food Safety and Nutrition that, among other measures, encourage manufacturers to reformulate and reduce the energy density and added sugar of food products [8]. This plan targets specific food subgroups such as sugar sweetened beverages, cakes and pastries and breakfast cereals, and suggests that added sugar reduction should reach around 10% by the end of 2020. By definition, LNCS are also referred to as artificial sweeteners, nonnutritive sweeteners, high-intensity sweeteners, and non-caloric sweeteners [9]. There are nineteen authorized LNCS in Europe: sorbitol (E-420), mannitol (E-412), acesulfame K (E-950), aspartame (E-951), cyclamate (E-952), isomalt (E-953), saccharine and its Sodium, Potassium and Calcium salts (E-954), sucralose (E-955), thaumatin (E-957), neohesperidine DC (E-959), steviol glycosides or “stevia” (E-960), neotame (E-961), salt of aspartame-acesulfame (E-962), polyglycitol syrup (E-964), maltitols (E-965), lactitol (E-966), xylitol (E-967), erythritol (E-968) and advantame (E-969) [10,11]. These food additives are used as substitutes because they have fewer calories (aspartame provides 4.0 kcal/g but it is 180 times sweeter than sugar [12]). Food composition databases and tables might not reflect these rapidly occurring changes in the food supply as most databases lack information on these ingredients in foodstuffs (presence and quantity), therefore their main dietary sources remain unknown [13]. Nutrition research requires comprehensive nutrient and component databases capable of capturing newly introduced or reformulated products in the marketplace [7,14]. Moreover, very few studies using comparable methodologies have been published at present.

As there is an increased consumer interest in reducing added sugars intake, food products including LNCS have become popular as a measure to control weight and to avoid other potential health adverse effects [15]. The role of LNCS in weight management is a still a subject of controversy as some researchers showed no benefit from their consumption and even weight gain, metabolic syndrome or type 2 diabetes [15,16,17]. A recent review from cross-sectional and prospective cohort studies evaluating the effects of LNCS on metabolism, weight, and obesity-related chronic diseases concluded that, although their consumption is associated with higher body weight and metabolic disease in observational studies, randomized controlled trials demonstrate that LNCS may support weight loss, especially when used in combination with behavioral weight loss support [18]. Therefore, detrimental effects remain under active discussion and underline the need to assess and monitor the consumption of these additives.

Research from the United States of America (USA), Australia, Mexico and Canada shows that, in their current food supply, LNCS are widely used in thousands of beverages such as fruit nectars, juice soft drinks, plant beverages, diet soft drinks and other food products such as desserts and dairy products, yoghurts and fermented milks, ice cream, jellies, candies, chewing gum, cakes, etc. [7,9,19]. Food manufacturers often use a blend of LNCS or a blend of sugars and LNCS to improve their flavor acceptability for reducing sugar intake [20,21].

Extensive scientific research has established the safety of all LNCS allowed for food use in the European Union. It is documented by the results of several in vitro and in vivo animal studies, tests in humans and in some cases epidemiological studies [12]. Furthermore, they have been evaluated through a risk assessment process covering hazard identification and characterization, exposure assessment and risk characterization [22]. All additives, including LNCS, have an Acceptable Daily Intake (ADI) level that represents a quantity guideline for health safety purposes; international regulatory bodies (i.e., Joint FAO/WHO Expert Committee on Food Additives) establish these levels. The ADI is defined as the amount of an authorized additive that can be consumed in a person’s daily diet (food or drink) over an entire lifetime without any appreciable risk to health. LNCS declaration in product labeling is mandatory [10,11] and, in addition, if aspartame is used as an ingredient, it is also required to declare the name and the phrase “a source of phenylalanine” on the labeling [10,23]. Beverages and specifically diet-carbonated beverages comprise the largest proportion of LNCS consumption worldwide, followed by tabletop non-caloric sweeteners and LNCS-containing foods [21]. Although added sugar intake was assessed in the ANIBES Study [3], the distribution and type of LNCS among food groups has not been studied due to the lack of information in Spanish food composition databases.

As their role in weight management and health remains a topic of continued debate, in 2014, a group of scientists signed the Chinchón declaration [24] which stated “the need to strengthen research on LNCS in Spain, to incentivize the monitoring of LNCS intake levels in different population groups and facilitate the execution of multidisciplinary projects on the subject”. More recently, in the Ibero–American Consensus on LNCS, a panel of worldwide experts, provided a comprehensive analysis and evaluation of the role of LNCS in food safety, their regulation and the nutritional and dietary aspects of their use in foods and beverages [25]. Amongst their conclusions, it was underlined that “LNCS are some of the most extensively evaluated dietary constituents, and their safety has been reviewed and confirmed by regulatory bodies globally including the WHO, the US Food and Drug Administration and the European Food Safety Authority” and that “consumer education about these products must be strengthened in a rigorous, objective way, based on the best scientific evidence and regulatory processes”. Regarding the relative safety of consumption of LNCS, international scientific experts in food, nutrition, dietetics, endocrinology, physical activity, pediatrics, nursing, toxicology and public health developed a consensus that emphasizes the long process of scientific risk assessment that it is demanded [25]. Regulatory bodies require data on reproductive and developmental toxicity, as well as mutagenicity/genotoxicity, carcinogenicity, immunotoxicity, neurotoxicity, from a battery of acute and chronic studies before a food additive can be considered for use [25]. Furthermore, the safety of approved compounds is continuously re-evaluated to consider new and relevant scientific data.

LNCS intakes are difficult to assess because manufacturers provide no labeling values of added quantities and consumption of LNCS is likely to be under-estimated [9]. Updated information regarding LNCS intake and distribution amongst food groups is limited in Spain and, to our knowledge, there is no monitoring or assessment of LNCS in foods and beverages sold and consumed in Spain. Thus, the aim of the present work was to identify the presence and types of added sugars and LNCS consumed through food groups according to the consumption patterns observed in the ANIBES study.

## 2. Materials and Methods 

### 2.1. Sample

**Figure 1:**
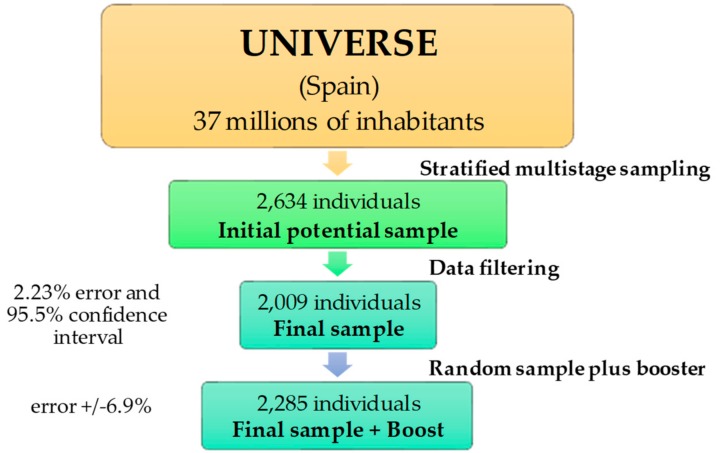
Flow diagram of sampling procedure.

The complete design, protocol, and methodology of the ANIBES study have been described in detail elsewhere [6]. In summary, the final sample of the study (*n* = 2009 individuals) presented an error of ±2.23% with a margin of confidence of 95.5% (1,013 men, 50.4%; 996 women, 49.6%; 2.23%). In addition, for the youngest groups (9–12, 13–17, and 18–24 years old), a boost was considered to at least *n* = 200 per age group, which increased the statistical power of the study (6.9% error) (Figure 1). The random sample was used to show total sample data and to compare between sexes; a booster sample was used to compare age groups and sex in age groups. Sample quotas according to the following variables were: age groups, sex, geographical distribution and locality size. The final fieldwork was carried out from mid-September to November (three months) 2013.

### 2.2. Food and Beverage Records

Study participants were provided with a tablet device (Samsung Galaxy Tab 27.0, Samsung Electronics; Suwon, Gyeonggi-do, Korea) to record, by writing descriptions and taking photographs, all food and drinks consumed for three days, both at home and outside, before and after each meal. Additionally, a brief description of the meals, recipes, brands, etc. could be included. Each participant completed a three-day food diary including one weekend day. The ANIBES in-house software was developed to receive information from the field tablets every two seconds, and the database was updated every 30 min. With the use of these photographs, and participant’s descriptions (i.e., cooking methods, type of oil, etc.), the dieticians/nutritionists codified the foods and beverages and assigned grams following three different cleanings of the data.

### 2.3. Selection of Brands

For each coded packaged product identified in the survey (*n* = 434) according to the selection of food groups of the ANIBES study [6], 2–7 food products from different brands, either traditional manufacturer’s brands or from distribution brand (supermarket own brand), were selected and photographed; being representative of at least >80% of the Spanish market, as a weighted average by sales. Photographs were taken in retail centers, such as hypermarkets, supermarkets and convenience stores by researchers, and comprised at least one, and up to four different shots to obtain precise information about packaging, company, brand, nutritional labeling and readable ingredient lists.

### 2.4. LCNS and Added Sugars Identification

From the ingredients lists depicted in the photographs, LNCS and added sugar were identified. There are nineteen authorized LNCS in Europe that were identified either by name or standardized code: sorbitol (E-420), mannitol (E-412), acesulfame K (E-950), aspartame (E-951), cyclamate (E-952), isomalt (E-953), saccharine and its Sodium, Potassium and Calcium salts (E-954), sucralose (E-955), thaumatin (E-957), neohesperidine DC (E-959), steviol glycosides or “stevia” (E-960), neotame (E-961), salt of aspartame-acesulfame (E-962), polyglycitol syrup (E-964), maltitols (E-965), lactitol (E-966), xylitol (E-967), erythritol (E-968) and advantame (E-969) [10,11]. In addition, added sugars were identified by the ingredient’s names that, in accordance to Regulation 1169/2011 of the European parliament and of the Council of 25 October 2011 on the provision of food information to consumers [26], includes all monosaccharides and disaccharides present in food, but excludes polyols. Since the purpose of this work was also identifying added sugar sources, the following terms were searched on labels: caramel, caramelized sugar, corn dextrose, dextrose, fructose, glucose syrup, glucose-fructose syrup, honey, inverted sugar, lactose and sugar (sucrose).

### 2.5. Collection of Food Composition for the Database

Food products were classified into groups and subgroups according to the ANIBES study categorization [6] (Table S1) to assess their LNCS and/or added sugar distribution.

Photographs were reviewed, and information was recorded for each food product in an in-house database built in Excel^®^ 2007 software (Microsoft Co., Redmond, Washington, DC, USA).

### 2.6. Statistical Analysis

Data were grouped into 16 food groups, 38 subgroups, and 761 ingredients for in-depth analysis. The results show the presence of LNCS or added sugars into different food groups, independent of their LNCS or added sugar content, and the mathematical calculations were done with Excel^®^ 2007 software (Microsoft Co., Redmond, Washington, DC, USA).

## 3. Results

The statistical description of the ANIBES sample population sample is included in Table 1. A total of 434 products were identified from consumption of participants of the ANIBES Study, and by including different brands (traditional vs. distribution), we compiled 1164 foods and beverages (3037 photographs).

From analyzed brands, 42% of products included some type of added sugars and 10% contained LNCS in their composition. Only 5.1% of the products had added sugars and LNCS declared on their labels. Table 2 shows the distribution of presence (%) of added sugars and LNCS in each of the food groups consumed.

**Figure 2:**
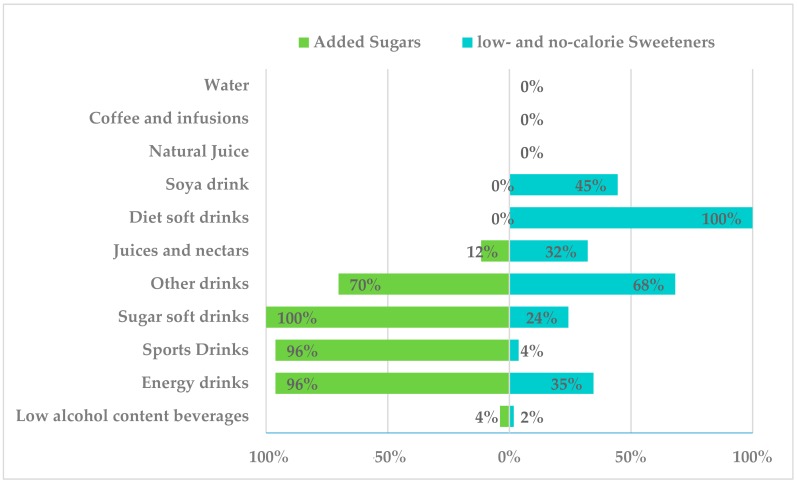
Presence of added sugar and low- and no-calorie sweeteners in beverages consumed by the Spanish ANIBES Study.

In the following figures, the presence of added sugars in different food subgroups is shown on the left hand side (independent of their LNCS content), while, on the right hand side, the presence of LNCS independently of sugar content for each food subgroup is presented. Figure 2 shows added sugar and LNCS content of non-alcoholic beverages. Water, coffee and infusions, and natural fruit juices had no added sugars or LNCS. Soya drinks included LNCS in 45% of assessed products. Diet soft drinks contained LNCS in 100% of assessed products, followed by “other drinks” (68%, alcohol-free biter, alcohol-free beer, juice, milk mixes, etc.). Noteworthy, 70% of the last group had added sugars. In turn, 100% of sugar soft drinks had added sugars and 24% LNCS, followed by energy drinks in which 96% of assessed products added sugars and 34% contained LNCS. Additionally, in the sports drinks category, 96% had added sugars but only 4% LNCS. Of all the juices and nectars assessed, 32% contained LNCS, whereas 12% contained added sugars. Finally, only 4% of the low alcohol content beverages present in the survey had added sugars (beers, bitter, sangria, cider and combined low-graded), whereas 2% contained only LNCS. Combined low alcohol content beverages presented LNCS in 76.7% of the assessed and 60% added sugars (data not shown). The major forms of added sugars in these groups were sucrose, glucose, fructose, glucose-fructose syrup and caramel. Main LNCS were acesulfame-k (E-950), aspartame (E-951), cyclamic acid or cyclamate (E-952), saccharine (E-954), sucralose (E-955) and neosperidine DC (E-959). Data from alcoholic beverages were not assessed, as ingredient information was unavailable on most product labels.

**Figure 3:**
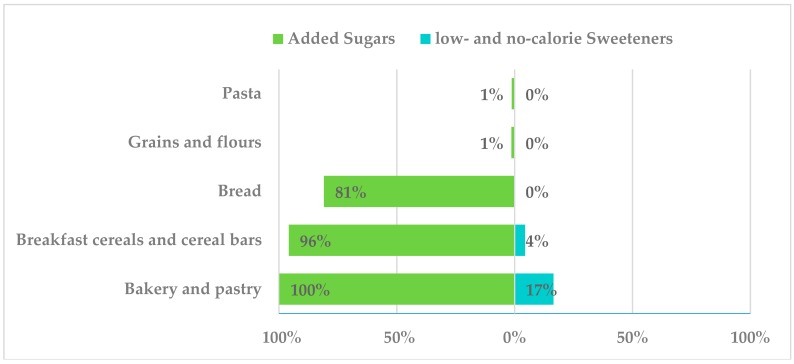
Presence of added sugars and low- and no-calorie sweeteners in grains consumed by the Spanish ANIBES population.

Within the “grains” group, all recorded bakery and pastry products had added sugars (Figure 3). These were mainly sugar, dextrose, glucose-fructose syrup, glucose syrup, inverted sugars, trehalose and caramel. However, only 17% of products contained LNCS such as sorbitol (E-420) (e.g., muffins, strudel and cakes) used at quantum satis level as a humectant.

Added sugars were present in 96% of breakfast cereals and cereal bars products, namely sugar, dextrose, glucose-fructose syrup, glucose syrup, inverted sugars, trehalose, corn glucose and fructose syrup and caramel. About only 4% from this group contained LNCS: mainly cereal bars, regular and whole, being E-420 the unique LNCS used at quantum satis levels for purpose other than sweetening.

Bread and bread products (derivatives) presented added sugars in 81% of assessed products (sugar, glucose-fructose syrup, inverted sugars and lactose). Interestingly, no LNCS were reported in this subgroup.

**Figure 4:**
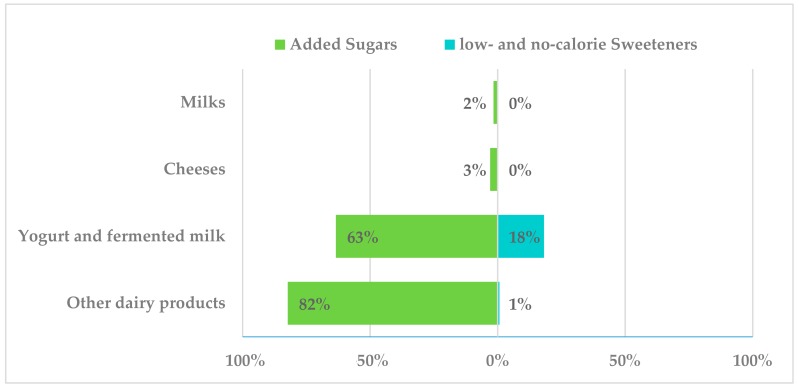
Presence of added sugars and low- and no-calorie sweeteners in milk and dairy products consumed by the Spanish ANIBES population.

Added sugars were present in 2% of milk products (Figure 4), specifically, in condensed milk. Similarly, cheeses contained added sugars in only 3% of reported products. None of these two groups contained LNCS. However, “other dairy products”, a subgroup that comprised milkshakes, dairy desserts and ice cream, reported 82% of products containing added sugars, namely, ice cream contained sugar in 100% of products (sugar, caramel, glucose-fructose syrup, lactose and dextrose). In addition, yogurt and fermented milk products had added sugars in a high proportion of reported products (63%, sucrose, glucose, fructose, lactose and glucose-fructose syrup), while 18% of products in this subgroup had LNCS, mainly acesulfame-k (E-950), aspartame (E-951), cyclamate (E-952) and sucralose (E-955).

**Figure 5:**
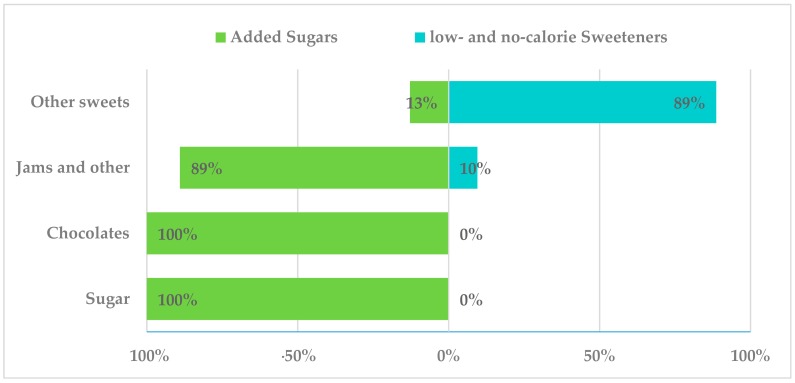
Presence of added sugars and low- and no-calorie sweeteners in sugar and sweets consumed by the Spanish ANIBES population.

In the “sugar” (table sugar) and “chocolates” group, 100% of products only contained added sugars (sucrose, honey, lactose and dextrose) and no LNCS were declared in their labels (Figure 5). Jams and others presented added sugars in 89% of products, namely sucrose and glucose-fructose syrup; conversely, LNCS were present in roughly 10% of this group (sorbitol (E-420), mannitol (E-421), acesulfame-k (E-950), aspartame (E-951), cyclamate (E-952), sucralose (E-955) and stevia (E-960)).

Other sweets, including candy, chewing gum, marzipan, licorice and “turron” (a traditional Spanish type of nougat) had added sugars in 13% of assessed products (sucrose, glucose-fructose syrup, honey and inverted sugars) while 89% had LNCS (sorbitol (E-420), mannitol (E-421), acesulfame-k (E-950), aspartame (E-951), cyclamate (E-952), saccharine (E-954), sucralose (E-955) and xylitol (E-967)).

**Figure 6:**
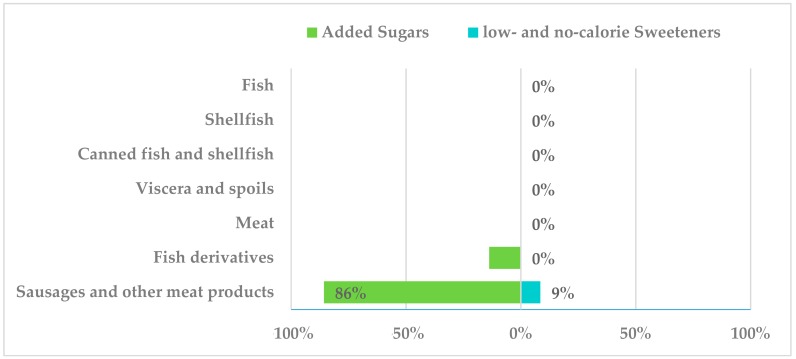
Presence of added sugars and low- and no-calorie sweeteners in meat and fish and derivate products consumed by the Spanish ANIBES population.

In the following group, “meat” and “fish” refers to unprocessed products (only refrigerated or chilled cuts). Sausages and other meat products and fish derivatives encompass those processed products that might contain ingredients other than meat in their composition. Presence of added sugars was observed in 86% of the sausages and other meat products subgroup (i.e., sausages, bacon, chorizo, etc.) and in 14% of fish derivatives (Figure 6), mainly sucrose, dextrose, lactose and glucose and dextrose syrup. LNCS were present in 8.5% of sausages and other meat products (sorbitol, E-420) but not in the second subgroup.

**Figure 7:**
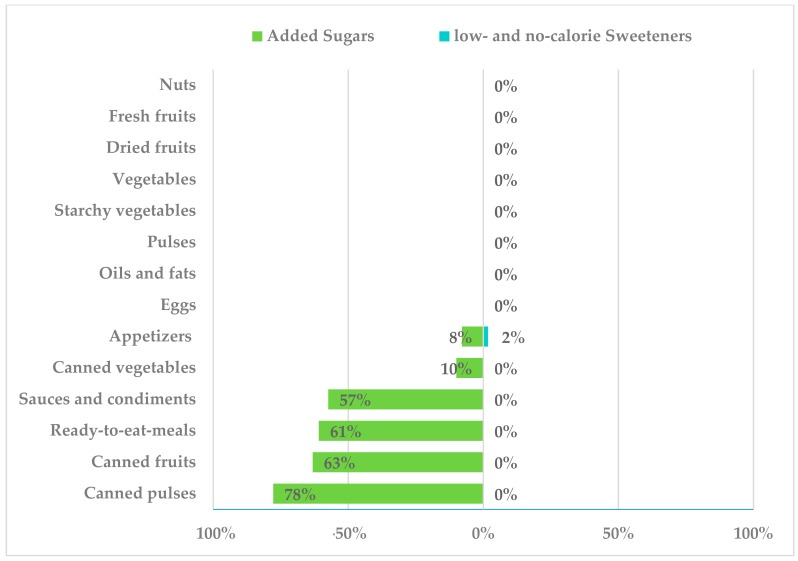
Presence of added sugars and low- and no-calorie sweeteners in other food groups consumed by the Spanish ANIBES population.

Canned pulses and fruits presented higher proportions of products containing added sugars, 78% and 63%, respectively, while only 10% of assessed canned vegetables did (Figure 7). As for “ready-to-eat meals”, we observed that 61% of assessed products had added sugars (sucrose, lactose, caramel, and corn dextrose and glucose syrup). Appetizers had added sugars in 8% of products (sucrose, glucose-fructose syrup and glucose syrup) and LNCS in 2% (aspartame, E-951). Finally, amongst sauces and condiments, 57% of assessed products had added sugars such as sucrose, caramel, lactose and glucose-fructose syrup.

Table 3 and Table 4 show the prevalence of the different types of added sugars and LNCS found, respectively. Overall, sucrose was the major added sugar ingredient (Table 2). Aspartame (E-951) and acesulfame K (E-950) were the most used LNCS across the different studied food groups, while neosperidine DC (E-959) was employed only in the non-alcoholic beverages category (Table 3).

## 4. Discussion

LNCS are widely used across many different types of food and beverage products commercialized in Spain. We examined a total of 1164 foods and beverage products grouped into 16 groups and 38 subgroups. To our knowledge, this is the first work conducted in our country to identify, examine and describe the presence of added sugars and LNCS in main food groups consumed by a representative sample of Spanish population. One of our main results is that 14 food subgroups declared including LNCS on their labels, of which eight belonged to the “beverages” group: soya drinks, diet soft drinks, juices and nectars, “other drinks”, sugar soft drinks, sports drinks, energy drinks and low-alcohol content beverages. In addition, breakfast cereals and cereal bars, bakery and pastry, yogurt and fermented milk, “other sweets”, jams and others, sausages and other meat products and appetizers.

In a recent publication by Dunford et al. [7], where LNCS were assessed in a total of 332,402 packaged branded food and beverage items across four countries (Australia, Mexico, New Zealand and the United States of America (USA)), authors also report that the “beverage” categories such as “soft drinks/sodas” had a higher proportion of products containing LNCS. However, in contrast with our observations, they found that “energy drinks” and “sports drinks” also presented this higher LNCS proportion, while in our study a higher proportion of these subgroups contained added sugars. It is useful to know which of the authorized LNCS and in what products they are employed, as well as if they are used alone or in combination with other LNCS or with added sugars. In this regard, in our sample, aspartame (E-951) and acesulfame K (E-950) were the most used LNCS, while neosperidine DC (E-959) was only utilized in the non-alcoholic beverages category. This is similar to findings from Norway [28], where the first two LNCS are also used in beverages. In turn, studies from Ireland [29] and Italy [30] reported sucralose and acesulfame K as the most commonly used sweeteners, respectively. Furthermore, both studies affirmed that intakes of each of these sweeteners by the total population were below the relevant ADI level [29,30]. In the USA, the trends in sales of LNCS show that the main sweeteners are aspartame and sucralose [9].

Added sugars were present in 25 subgroups (six main food groups) in our study, clearly a higher proportion than the inclusion of LNCS. These were mainly found in the “beverages” group, “milk and dairy products”, “sugar and sweets” and “other food groups”. In Canada, Acton et al. [31] analyzed 40,000 processed food products available for sale in 2015 and found that 66% contained at least one type of added sugar. In the USA, it was estimated that 68% of available processed foods and beverages contain caloric sweeteners (i.e., added sugars), 74% comprise both caloric and LNCS and 5% include only LNCS [32]. An Australian study, which examined over 5744 packaged foods, found that added sugar was present in 61% of the sample and LNCS ingredients had an even higher presence (69%). Moreover, only 31% of foods had no added sugars or LNCS. Sucrose, glucose syrup, maple syrup, maltodextrin and glucose/dextrose were the most common sugar ingredient types identified [33]. Lower proportions were found in Slovenia, where researchers evaluated 10,674 pre-packaged food items and found 52.6% contained added sugar [34]. Although several international studies undertook the assessment of LCNS and added sugar content in food products, most available food composition tables and databases still lack the detailed ingredients used in processed food and beverage products. Therefore, LNCS are not included neither added sugars are specified as such. This is also the case in Spain, where the present work could be a useful starting point for completing and updating these tables and databases through a harmonized data interchange with the research groups in charge of their management.

In terms of the potential public health impact in our country, it is relevant to identify LNCS in food and beverage products as the European regulation establishes that their consumption should be monitored and quantified in different population groups. At present, there are no published results on this matter for the Spanish population. In addition, added sugars contents should be reduced to meet public health guidelines and key products that contain them could be monitored for reformulation as well.

There are several general limitations affecting the precision and reliability of dietary assessments, such as the use of self-report methods, underreporting and reliance on participant’s memory, amongst others. In the ANIBES Study, the decision to use a three-day register for the estimation of the intake was due to the precision of the method, considered as the reference method for it [35]. A common limitation from this method is that the individual must know how to read and write, an aspect that fortunately today is fulfilled by practically the entire population. The limitations such as the time spent and cooperation of the participant, in addition to the cost of coding and analysis can be reduced thanks to the use of new technologies. Precision can be affected because the participant feels monitored and can change their habits. For that reason, it was decided to carry out this registration for three days, since it has been seen that fewer days may not be representative and more than 4–5 could affect the quality of the data, since the participant is discouraged and participation decreases [35].

One of the limitations from the ANIBES Study is the cross-sectional design, which provides evidence for association but not for casual relationship. There are also additional shortcomings in the assessment of LNCS consumption related to the presence and description of specific types of LNCS on product’s labels. For instance, the use of the “E-xxx” code that identifies a particular LNCS might be misleading for consumers and untrained dietitians and remain undetected. This can also be the case when evaluating added sugars as they are listed the ingredients list under many denominations. Furthermore, added sugar from high-alcoholic beverages were not studied, as manufacturers are not required to declare them. Therefore, many products that contain LNCS or added sugars may be overlooked in dietary surveillance. For example, the United States Department of Agriculture (USDA) Food and Nutrient Database for Dietary Studies (FNDDS) and the USDA National Nutrient Database for Standard Reference include many food and beverage items as either containing a “low- and non-calorie sweetener”, being “dietetic”, or “sugar-free”, however, there is a lack of information regarding the specific LNCS used [9].

Amongst the main strengths of our work is the use of the ANIBES study, a nationally representative sample that includes seven geographical zones (Nielsen zones [6]), three habitat size classes (urban, semi-urban and rural) and different age and socio-economical segments. This allowed us to focus on food categories that are representative of foods most commonly consumed. Secondly, the use of the tablet devices by participants allowed the detailed compilation of relevant products that presented LNCS and added sugars, overcoming the problem of underestimation or forgetting. Tablet devices, as well as smartphones, are being increasingly used by population for work or leisure, and some studies found them to be very useful for recording dietary intakes and self-monitoring [36]. Finally, the survey designed to collect product’s labels, including traditional as well as distribution brands, added reliability and representativity to the data collection.

## 5. Conclusions

The present work shows that currently there is a widespread presence of LNCS and added sugars in many food and beverage groups commercialized in Spain. LNCS were present in diet soft drinks (100%), “other sweets” (89%), soya drinks (45%), and yogurt and fermented milks (18%). Added sugars were present in sugar soft drinks (100%), energy drinks (96%), sports drinks (96%), bakery and pastry (100%), chocolates (100%), ice cream (100%), breakfast cereals/bars (96%) and jams (89%). Main LNCS were acesulfame K, aspartame, cyclamate and sucralose. Sucrose, dextrose, glucose-fructose syrup, caramel and honey were the main added sugars.

It is relevant to identify LNCS in food and beverage products as the European regulation establishes that their consumption should be monitored and quantified in different population groups. Moreover, added sugars contents should be reduced to meet public health guidelines and key products that contain them could be monitored for reformulation as well. This is the first work conducted in Spain to identify, examine and describe the presence of added sugars and LNCS in main food groups consumed by a representative sample of our population. This information should be compiled in food composition databases to assess their consumption in nutritional surveys and monitor reformulation.

## Figures and Tables

**Table 1:** Statistical description of the sample ANIBES (Modified from Nissensohn et al., 2016 [27]).

		Total	%	Male	%	Female	%
		2007	100	1011	50.4	996	49.6
Age Group	9–12	100	5	62	6.1	38	3.8
	13–17	123	6.1	84	8.3	39	3.9
	18–39	777	38.7	387	38.3	390	39.2
	40–64	810	40.4	385	38.1	425	42.7
	65–75	197	9.8	93	9.2	104	10.4
Level of education	Primary or less	743	37	378	37.4	365	36.6
	Secondary	858	42.8	434	42.9	424	42.6
	Tertiary or University	406	20.2	199	19.7	207	20.8
Economical level	1000 € or less	397	19.8	191	18.9	206	20.7
	From 1000 to 2000 €	795	39.6	393	38.9	402	40.4
	Over 2000 €	320	15.9	163	16.1	157	15.8
	No income	7	0.3	4	0.4	3	0.3
	No answer	488	24.3	260	25.7	228	22.9
Geographical distribution	Northwest	152	7.6	77	7.6	75	7.5
	North Central	161	8	79	7.8	82	8.2

**Table 2:** Presence of added sugars and low- and no-calorie sweeteners (LNCS) across the food groups consumed in the ANIBES Study.

Food Groups	Presence of Added Sugars		Presence of LNCS		Presence of Added Sugars and LNCS
	No	Yes	No	Yes	
Appetizers (*n* = 19)	*n* = 15; 79%	*n* = 4; 21%	*n* = 18; 95%	*n* = 1; 5%	*n* = 1; 5%
Sugars and sweets (*n* = 75)	*n* = 12; 16%	*n* = 63; 84%	*n* = 64; 85%	*n* = 11; 15%	*n* = 1; 1%
Low alcohol content beverages (*n* = 52)	*n* = 22; 71%	*n* = 9; 29%	*n* = 27; 87%	*n* = 4; 13%	*n* = 2; 6%
Non-alcoholic beverages (*n* = 148)	*n* = 94; 64%	*n* = 54; 36%	*n* = 90; 61%	*n* = 58; 39%	*n* = 22; 15%
Meat and meat products (*n* = 92)	*n* = 52; 57%	*n* = 40; 43%	*n* = 89; 97%	*n* = 3; 3%	*n* = 3; 3%
Cereals/grains (*n* = 241)	*n* = 87; 36%	*n* = 154; 64%	*n* = 230; 95%	*n* = 11; 5%	*n* = 11; 5%
Fruits (*n* = 83)	*n* = 79; 95%	*n* = 4; 5%	*n* = 83; 100%	*n* = 0; 0%	*n* = 0; 0%
Eggs (*n* = 8)	*n* = 8; 100%	*n* = 0; 0%	*n* = 8; 100%	*n* = 0; 0%	*n* = 0; 0%
Milk and dairy products (*n* = 299)	*n* = 135; 45%	*n* = 164; 55%	*n* = 262; 88%	*n* = 37; 12%	*n* = 24; 8%
Pulses (*n* = 23)	*n* = 20; 87%	*n* = 3; 13%	*n* = 23; 100%	*n* = 0; 0%	*n* = 0; 0%
Fish and shellfish (*n* = 96)	*n* = 95; 99%	*n* = 1; 1%	*n* = 96; 100%	*n* = 0; 0%	*n* = 0; 0%
Ready-to-eat meals (*n* = 65)	*n* = 28; 43%	*n* = 37; 57%	*n* = 65; 100%	*n* = 0; 0%	*n* = 0; 0%
Sauces and condiments (*n* = 49)	*n* = 76; 69%	*n* = 34; 31%	*n* = 110; 100%	*n* = 0; 0%	*n* = 0; 0%
Vegetables (*n* = 76)	*n* = 73; 96%	*n* = 3; 4%	*n* = 76; 100%	*n* = 0; 0%	*n* = 0; 0%

**Table 3:** Prevalence of type of added sugars declared on food products with added sugar consumed by the ANIBES study population.

Added Sugars	Prevalence
Sugar (sucrose)	50.3%; *n* = 308
Sugar (sucrose) and glucose-fructose syrup	5.7%; *n* = 35
Sugar (sucrose) and glucose syrup	4.1%; *n* = 25
Dextrose	2.8%; *n* = 17
Fructose	2.8%; *n* = 17
Lactose	2.6%; *n* = 16
Sugar (sucrose) and lactose	2.5%; *n* = 15
Sugar (sucrose) and dextrose	2.3%; *n* = 14
Sugar (sucrose) and caramel	2.0%; *n* = 12
Caramel	1.8%; *n* = 11
Glucose-fructose syrup	1.1%; *n* = 7

Only those added sugars which contribute to at least 1% of intakes have been included, calculated as percentage of assessed products containing added sugars.

**Table 4:** Prevalence of type of Low- and no-calorie sweeteners (LNCS) declared on food products with LNCS consumed by the ANIBES study population.

Low- and No-Calorie Sweeteners	Prevalence
Acesulfame K (E-950)	30.5%; *n* = 80
Sucralose (E-955)	30.2%; *n* = 79
Aspartame (E-951)	10.7%; *n* = 28
Cyclamate (E-952)	10.7%; *n* = 28
Sorbitol (E-420)	7.3%; *n* = 19
Saccharine and its Sodium (E-954)	6.1%; *n* = 16
Neohesperidine DC (E-959)	1.5%; *n* = 4
Mannitol (E-412)	1.1%; *n* = 3
Steviol glycosides or “stevia” (E-960)	1.1%; *n* = 3
Thaumatin (E-957)	0.4%; *n* = 1
Xylitol (E-967)	0.4%; *n* = 1

## Supplementary Materials

The following are available online at http://www.mdpi.com/2072-6643/10/9/1265/s1, Table S1: Food group classification and included subgroups assessed in the ANIBES Study (Adapted from Pérez-Rodrigo et al. (1)).
