# Supplementary material for: Added Sugars and Low- and No-Calorie Sweeteners in a Representative Sample of Food Products Consumed by the Spanish ANIBES Study Population
Source: Nutrients. 2018 Sep 7;10(9):1265. doi: 10.3390/nu10091265 (PMC6163363; doi:10.3390/nu10091265)
Supplement: Supplementary file 1 [file nutrients-10-01265-s001.docx]

**Supplementary Material**

**Table S1. Food group classification and included subgroups assessed in the ANIBES Study**

(Adapted from Pérez-Rodrigo et al. (1))

| **Food group** | **Included subgroups** |
| --- | --- |
| **Cereals/Grains** | Grains and flours |
|  | Bread |
|  | Breakfast cereals and cereal bars |
|  | Pasta |
|  | Bakery and pastry |
| **Vegetables** | Including starchy tubers (i.e. potatoes) |
| **Fruits** |  |
| **Oils and fats** | Olive oil |
|  | Other oils |
|  | Butter, margarine and shortening |
| **Milk and dairy products** | Milk |
|  | Cheese |
|  | Yogurt and fermented milk |
|  | Other dairy products |
| **Fish and Shellfish** | Fish  Shellfish  Canned fish and shellfish  Fish derivatives |
| **Meat and meat products** | Meat  Sausages and other meat products Viscera and spoils |
| **Eggs** |  |
| **Pulses** |  |
| **Sugars and sweets** | Sugar |
|  | Chocolates |
|  | Jams and other |
|  | Other sweets |
| **Appetizers** |  |
| **Ready-to-eat-meals** |  |
| **Sauces and condiments** |  |
| **Non-alcoholic beverages** | Water |
|  | Coffee and infusions |
|  | Sugar soft drinks |
|  | Non-sweetened soft drinks |
|  | Sports Drinks |
|  | Energy drinks |
|  | Juices and nectars |
|  | Other drinks |
| **Alcoholic beverages** | Low alcohol content beverages |
|  |  |

1. Pérez-Rodrigo C, Gianzo-Citores M, Gil Á, González-Gross M, Ortega R, Serra-Majem L, et al. Lifestyle Patterns and Weight Status in Spanish Adults: The ANIBES Study. Nutrients. 2017;9 (6):606.
